# Latissimus Dorsi Transfer With Interposed Autograft for Axillary Nerve Palsy After Reverse Shoulder Arthroplasty: A Case Report

**DOI:** 10.7759/cureus.101459

**Published:** 2026-01-13

**Authors:** Nikolaos P Sachinis, Eleni Karagergou, Menelaos Mountzouris, Alexandros Givissis, Panagiotis Givissis

**Affiliations:** 1 First Orthopaedic Department of Aristotle University of Thessaloniki, General Hospital "G. Papanikolaou", Thessaloniki, GRC; 2 Orthopaedic Department, School of Medicine, European University of Cyprus, Nicosia, CYP

**Keywords:** axillary nerve palsy, deltoid paralysis, latissimus dorsi transfer, muscle transfer, reverse total shoulder arthroplasty

## Abstract

Persistent axillary nerve palsy after reverse shoulder arthroplasty (RSA) results in profound deltoid dysfunction and loss of active shoulder elevation. Traditional reconstructive options such as nerve repair or transfer may not be feasible in chronic cases. Latissimus dorsi tendon transfer augmented with an interposed graft has been described to restore shoulder abduction in deltoid-deficient shoulders, but its application after RSA is not well documented.

We present the case of a 73-year-old female patient who developed chronic axillary nerve palsy following RSA performed for complex proximal humerus fracture. Despite exhaustive conservative and rehabilitative efforts over 18 months, active elevation remained severely limited, and electromyography confirmed irreversible nerve injury. Given the deltoid-dependent biomechanics of RSA, functional reconstruction was pursued.

The patient underwent latissimus dorsi tendon transfer augmented with a semitendinosus autograft routed beneath the acromion and fixed to the deltoid tuberosity using suture anchors. This aimed to recreate a deltoid-like lever arm to restore active elevation. At 12 months postoperative follow-up, the patient demonstrated improved active shoulder elevation (up to 80°), enhanced stability, and increased functional independence in activities of daily living, with no significant complications.

Latissimus dorsi transfer with interposed autograft may serve as a viable reconstructive strategy to improve shoulder elevation and function in selected patients with chronic axillary nerve palsy after RSA. Further studies are needed to better define indications and long-term outcomes.

## Introduction

Loss of deltoid function leads to a profound deficit in active shoulder elevation. This impairment becomes particularly disabling in patients treated with reverse shoulder arthroplasty (RSA), where shoulder elevation depends primarily on deltoid activation rather than rotator cuff function [1].

Chronic axillary nerve palsy results in irreversible denervation and atrophy of the deltoid muscle, and in delayed presentations nerve reconstruction techniques are unlikely to restore meaningful deltoid function due to the time-dependent nature of motor reinnervation [2]. When neurological recovery is not expected, surgical strategies shift from nerve-based reconstruction to procedures that substitute deltoid function. The concept of replacing deltoid activity with a transferred muscle was first described by Itoh et al., who reported restoration of active shoulder elevation following transfer of the latissimus dorsi to replace a paralysed anterior deltoid in patients with axillary nerve injury [3]. Subsequently, De Smet reported successful reconstruction of shoulder abduction using a latissimus dorsi flap sutured to the anatomical deltoid insertion in a patient with irreversible axillary nerve palsy, demonstrating the feasibility of the latissimus dorsi as a functional deltoid substitute [4].

More recently, Moharram et al. described a refined latissimus dorsi tendon transfer technique augmented with a semitendinosus graft and routed over the acromion to recreate a deltoid-like lever arm. In their series of 10 patients with deltoid paralysis, mean active shoulder abduction improved from 27° preoperatively to 110° postoperatively, with a mean gain of 83° at a mean follow-up of 25.4 months [5]. The application of muscle transfer strategies in the specific setting of RSA remains limited. Elhassan et al. reported outcomes of RSA combined with pedicled pectoralis major transfer in patients with deltoid paralysis, demonstrating improvement in active forward elevation from a mean of 15° preoperatively to 83° postoperatively, as well as significant improvement in patient-reported outcomes at a mean follow-up of 37 months [1].

Despite these reports, there is no established reconstructive strategy for managing chronic axillary nerve palsy following RSA. The present case describes the adaptation of a latissimus dorsi tendon transfer augmented with an interposed autograft, based on previously described deltoid substitution principles, to restore shoulder elevation and stability in a patient with chronic axillary nerve palsy after RSA [5].

## Case presentation

A 73-year-old female patient presented with severe functional impairment of the shoulder in the outpatient upper limb clinic of our institution. She was right-hand dominant and had a medical history of arterial hypertension and hypothyroidism. She was retired at the time of presentation. Two years prior to presentation, the patient sustained a comminuted proximal humerus fracture that was treated at another institution with RSA. Postoperatively, she experienced persistent inability to actively abduct or elevate the shoulder. Despite prolonged conservative management and physiotherapy, no functional improvement was observed over time. Progressive atrophy of the deltoid muscle became clinically evident, and no signs of neurological recovery were noted.

On physical examination, passive range of motion of the shoulder was not fully preserved (forward elevation 120°, abduction 90°, internal rotation up to sacrum, external rotation 40°) but was painless. Active shoulder abduction was limited to approximately 20°, and active forward flexion did not exceed 40°. Active internal and external rotation were absent or minimal. Marked deltoid muscle atrophy was visible on inspection. Sensory examination revealed hypoesthesia over the lateral aspect of the shoulder.

**Figure 1:**
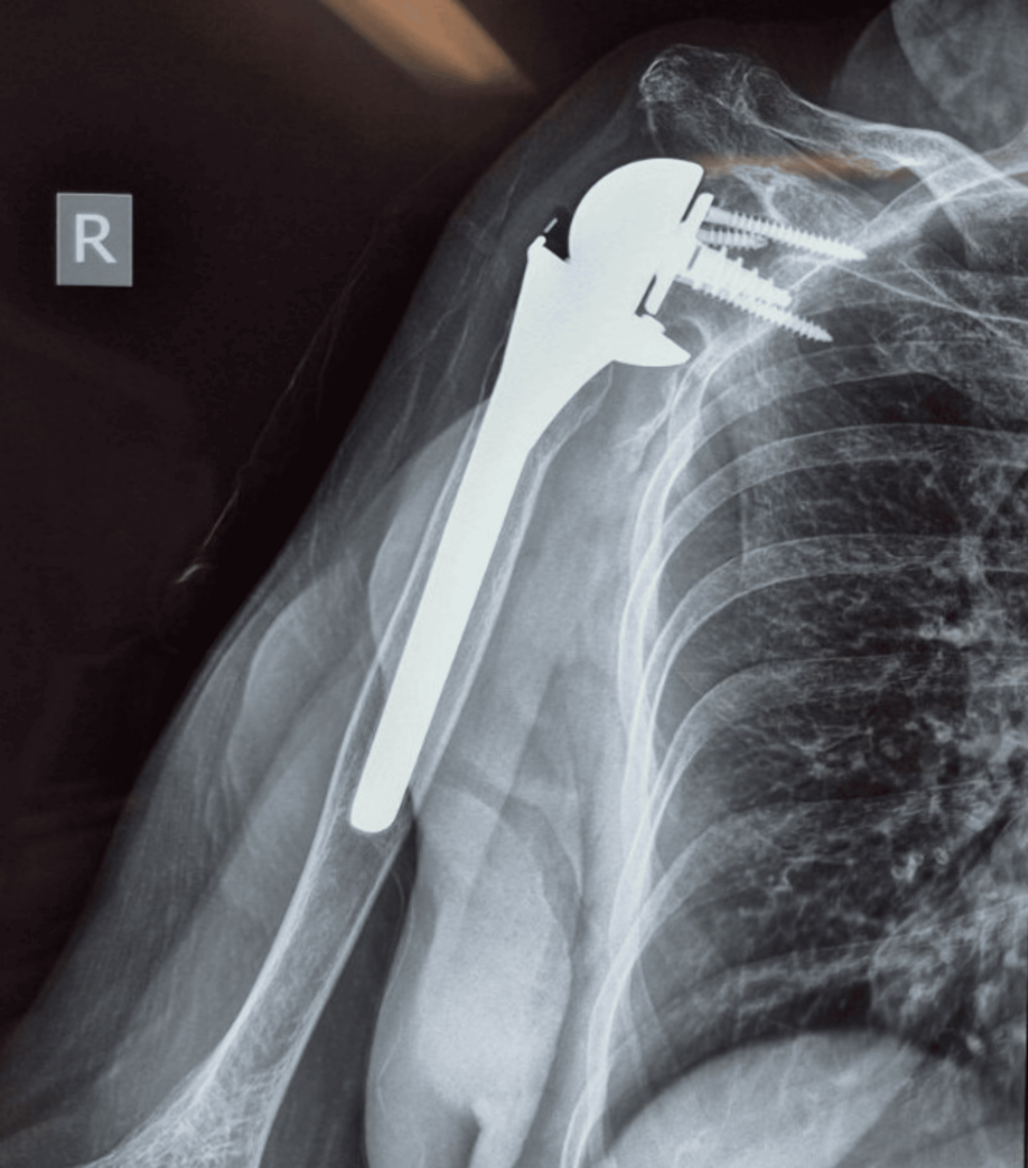
Anteroposterior radiograph of the right shoulder demonstrating a well-positioned reverse shoulder arthroplasty without signs of loosening, instability, or mechanical failure.

Standard radiographs demonstrated a well-positioned RSA, without signs of loosening, instability, or mechanical failure (Figure 1).

Electrophysiological evaluation confirmed chronic axillary nerve palsy (complete axon denervation) with no evidence of reinnervation of the deltoid muscle.

Given the chronicity of the axillary nerve palsy and the absence of neurological recovery two years after the index procedure, nerve repair or transfer was considered unlikely to restore deltoid function. Alternative reconstructive options were discussed. Shoulder arthrodesis was considered a last-resort salvage option. After multidisciplinary discussion and informed consent, a muscle transfer strategy was selected. A latissimus dorsi tendon transfer augmented with an interposed autograft was planned to functionally substitute the paralyzed deltoid muscle, adapted from the recent article of Moharram et al. [5]. 

**Figure 2:**
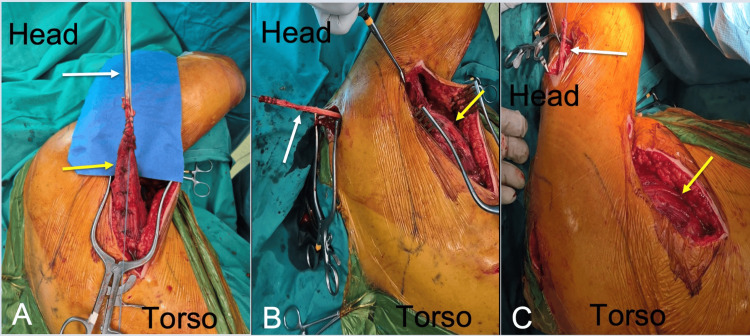
Intraoperative photographs illustrating the key steps of the latissimus dorsi tendon transfer augmented with an interposed autograft. Orientation markers indicate the direction of the head and torso. (A) Identification of the latissimus dorsi muscle (yellow arrow) and detachment of its insertion to the humerus through a posterior approach; the harvested semitendinosus tendon (white arrow) is sutured to the latissimus dorsi tendon by a side by side technique. (B) Passage of the interposed semitendinosus tendon (white arrow) beneath the posterior deltoid and posterior to the acromion. The latissimus dorsi muscle is indicated with a yellow arrow. (C) Final routing of the interposed semitendinosus tendon (white arrow) toward the deltoid tuberosity prior to fixation with the sutures of the placed metallic anchor (not shown). The latissimus dorsi muscle is indicated with a yellow arrow.

An autograft was harvested from the ipsilateral semitendinosus tendon. Through a posterior approach, the latissimus dorsi tendon was identified and detached from its humeral insertion. The harvested tendon graft was used to augment the latissimus dorsi tendon using a side-to-side suture technique. The augmented tendon was routed beneath the posterior portion of the deltoid and passed posterior to the acromion. Fixation was achieved at the deltoid tuberosity using a 4.5-mm metallic suture anchor. Intraoperative photographs (Figure 2) illustrate the key steps of the procedure.

**Figure 3:**
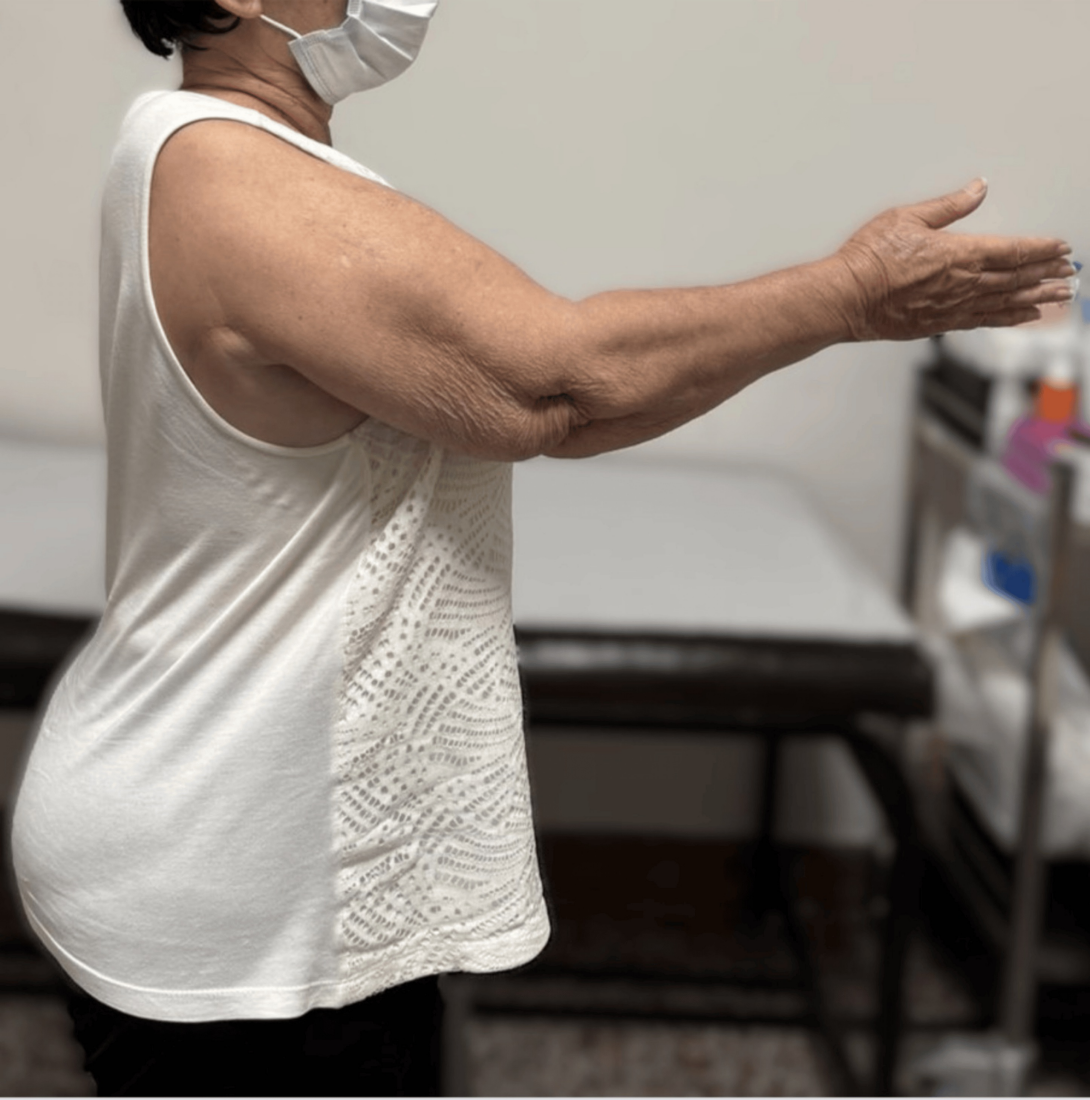
Clinical photograph at one-year follow-up demonstrating active forward elevation of the operated shoulder following latissimus dorsi transfer.

**Figure 4:**
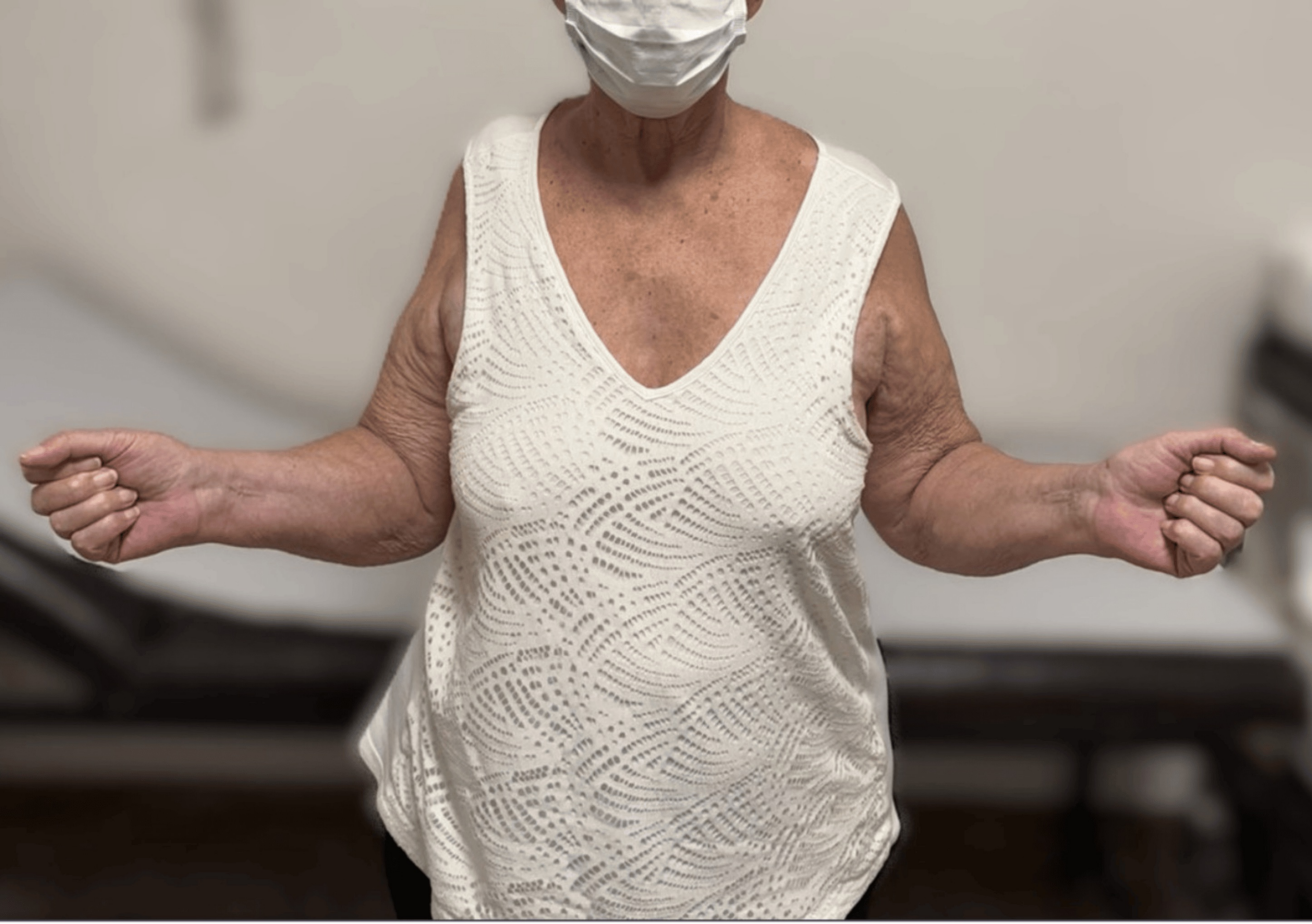
Clinical photograph at one-year follow-up demonstrating active external rotation of the operated shoulder, with improved control and stability compared to preoperative status.

Postoperatively, the shoulder was immobilized in an abduction brace for six weeks. Passive range-of-motion exercises were initiated during this period, followed by gradual active-assisted mobilization and strengthening. At one-year follow-up, the patient demonstrated active shoulder abduction of approximately 80° and improved forward elevation, with enhanced shoulder stability and increased independence in activities of daily living (Figures 3, 4). No perioperative or postoperative complications were observed.

## Discussion

Axillary nerve palsy represents a major challenge, particularly in patients treated with RSA, where active shoulder elevation depends almost entirely on deltoid function. As in our case, in delayed presentations, the absence of meaningful deltoid reinnervation necessitates consideration of alternative reconstructive strategies based on realistic functional expectations.

Nerve transfer procedures have demonstrated favorable outcomes when performed early after axillary nerve injury. Leechavengvongs et al. reported that triceps-to-axillary nerve transfer restored deltoid strength to Medical Research Council (MRC) grade M4 in all seven patients, with a mean active shoulder abduction of 124° at a mean follow-up of 20 months [2]. Similarly, Miyamoto et al. reported mean postoperative shoulder abduction of approximately 112° following nerve transfer, with near-universal recovery of deltoid strength [6]. However, both studies emphasized that outcomes were contingent on early intervention before irreversible muscle denervation and fatty infiltration, limiting the applicability of nerve-based reconstruction in chronic cases such as the present one, where complete deltoid paralysis persisted two years after RSA.

Muscle transfer procedures have therefore been proposed to substitute deltoid function when nerve reconstruction is no longer feasible. Itoh et al. were the first to describe transfer of the latissimus dorsi to replace a paralysed anterior deltoid, establishing the biomechanical feasibility of this concept [3]. De Smet subsequently reported restoration of shoulder abduction using a latissimus dorsi flap sutured to the deltoid insertion in a patient with irreversible axillary nerve palsy, confirming that the latissimus dorsi could functionally substitute the deltoid [4].

More robust quantitative data were provided by Moharram et al., who described a refined latissimus dorsi tendon transfer augmented with a semitendinosus graft and routed over the acromion to recreate a deltoid-like lever arm. In their series of 10 patients with deltoid paralysis, mean active shoulder abduction improved from 27° preoperatively to 110° postoperatively, representing a mean gain of 83°, at a mean follow-up of 25.4 months [5]. Forward flexion improved to a mean of 120.7°, and abduction strength reached approximately 62% of the contralateral side. These data provide a realistic benchmark for functional recovery following latissimus dorsi-based deltoid substitution.

Other muscle transfer options for deltoid paralysis have also been reported. Lin et al. described transfer of the superior portion of the pectoralis major for restoration of shoulder abduction, reporting improvement in active abduction from a mean of 6° preoperatively to 74° postoperatively and improvement in forward flexion from 10° to 75° at a mean follow-up of six years [7]. Trapezius transfer has likewise been proposed as a deltoid substitute. Kotwal et al. reported mean abduction gains of approximately 60° following trapezius transfer for deltoid paralysis, with good or excellent outcomes in around 60% of patients, although fixation-related complications were noted [8]. Ruhmann et al. further demonstrated that outcomes following trapezius transfer correlated with residual scapular control and preoperative muscle power, underscoring the importance of patient selection [9].

Evidence supporting muscle transfer specifically in the context of RSA remains limited but is growing. Elhassan et al. reported outcomes of RSA combined with pedicled pectoralis major transfer in 31 patients with deltoid paralysis [1]. At a mean follow-up of 37 months, mean active forward elevation improved from 15° preoperatively to 83° postoperatively. Patient-reported outcomes also improved, with the Subjective Shoulder Value increasing from 7% to 53% and the Disabilities of the Arm, Shoulder, and Hand (DASH) score improving from 54 to 33. Although complications such as acromial fractures occurred in a small number of patients, this study demonstrated that muscle substitution can restore meaningful function and stability in deltoid-deficient shoulders treated with RSA. Kermarrec et al. reported similar improvements in active elevation, typically reaching 80-90°, following RSA combined with various muscle transfer strategies in patients with deltoid palsy, further supporting the feasibility of this approach [10].

When reconstructive options are limited, shoulder arthrodesis has been proposed as a salvage procedure. Atlan et al. reported that more than 75% of patients with brachial plexus palsy achieved active shoulder abduction greater than 45° following glenohumeral fusion [11]. However, this procedure sacrifices glenohumeral motion and is associated with substantial functional limitations, making it less desirable in patients with preserved passive motion and a desire to regain active elevation.

In the present case, adaptation of a latissimus dorsi tendon transfer augmented with an interposed autograft resulted in restoration of active shoulder abduction to approximately 80° at one-year follow-up. This outcome is consistent with the lower range of abduction gains reported by Moharram et al. and comparable to functional improvements reported following muscle transfer procedures combined with RSA [1,4,5]. Although conclusions are limited by the single-case design, these findings suggest that latissimus dorsi-based deltoid substitution may represent a viable reconstructive option for carefully selected patients with chronic axillary nerve palsy following RSA.

## Conclusions

Chronic axillary nerve palsy following RSA presents a complex reconstructive problem due to irreversible deltoid dysfunction and limited potential for neurological recovery. This case demonstrates that latissimus dorsi tendon transfer augmented with an interposed autograft can partly restore active shoulder elevation and provide shoulder stability in a carefully selected patient with chronic axillary nerve palsy after RSA. Although general deductions are limited by the single-case design, the functional improvement observed at one-year follow-up is consistent with outcomes of other studies reporting deltoid substitution and muscle transfer procedures. Further studies with larger cohorts and longer follow-up are needed to better define indications and expected outcomes for this reconstructive strategy.
